# Striking differences in frost hardiness and inability to cold acclimate in two *Mougeotia* species (Zygnematophyceae) from alpine and lowland habitats

**DOI:** 10.1111/ppl.14167

**Published:** 2024-01-22

**Authors:** Charlotte Permann, Matthias Stegner, Thomas Roach, Valentina Loacker, Louise A. Lewis, Gilbert Neuner, Andreas Holzinger

**Affiliations:** ^1^ Department of Botany University of Innsbruck Innsbruck Austria; ^2^ Department of Ecology and Evolutionary Biology University of Connecticut Storrs CT USA

## Abstract

Zygnematophyceae, a class of freshwater green algae, exhibit distinctive seasonal dynamics. The increasing frequency of cold snaps during the growing season might challenge the persistence of some populations. The present study explored the frost hardiness of two *Mougeotia* species isolated from different elevations and habitats. Additionally, a phylogenetic (*rbc*L sequence), ultrastructural and physiological characterization was performed. Both species, grown under standard culture conditions and cold acclimated cultures (+4°C), were exposed to freezing temperatures down to −9°C. Furthermore, ultrastructural‐, hydrogen peroxide (H_2_O_2_)‐ and photosynthetic pigment analysis were performed on cells exposed to −2°C, with and without induced ice nucleation. The alpine *M. disjuncta* showed a higher frost hardiness (LT_50_ = −5.8°C), whereas the lowland *M. scalaris* was susceptible to ice. However, frost hardiness did not improve after cold acclimation in either species but rather decreased significantly in *M. disjuncta* (LT_50_ = −4.7°C). Despite darkness, prolonged sub‐zero temperatures or freezing induced the activation of the xanthophyll (VAZ) cycle in *M. scalaris*. Our results demonstrate that frost hardiness varies within the genus *Mougeotia* and that the VAZ cycle can be activated in the dark under subzero temperature‐ and freezing stress but does not necessarily increase frost hardiness. As highly frost hardy cell types are usually formed at the end of the growing season, the ability of young cells to survive ice formation in the upper subzero temperature range represents a crucial survival strategy in populations exposed to late spring frosts.

## 1 INTRODUCTION

Over the last few decades, the impact of climate change on plants has increased drastically, challenging the existence of ecosystems worldwide. While a rise in annual air temperature and severe droughts are the most prevalent effects, the general increase in weather extremes represents a major threat to plant communities. More frequent and severe cold snaps and freezes can have major impacts on plant development, especially during late spring (Schwartz et al., 2002; Gu et al., 2008; Marino et al., 2011; Chamberlain & Wolkovich, 2021). Freezing stress can cause serious membrane damage, especially to the plasma membrane, which is supposed to be the critical point of freeze–thaw damage (Arora, 2018). To survive such frost events and avoid damages caused by frost, plants have to manage ice formation, propagation and accommodation, as well as ice‐induced freeze dehydration. Important adaptations to ice management include changes in membrane lipids, sugars, osmolytes, proteins, genes, and transcription factors (Pearce, 1999; Thomaswhow, 1999; Knight & Knight, 2012; Wisniewksi et al., 2014), as well as cell wall structure/composition and leaf anatomy (Panter et al., 2019; Takahashi et al., 2021; Liu et al., 2022; Stegner et al., 2022, 2023). Recent data also suggest the involvement of the de‐epoxidation state (DEPS) of the xanthophyll cycle in the tolerance to desiccation and freezing stress (Fernández‐Marín et al., 2018, 2020, 2021a). Activation of the xanthophyll cycle and the enzymatic conversion of violaxanthin (V) to antherxanthin (A) and zeaxanthin (Z) is a ubiquitous response of photosynthetic organisms to high light. Zeaxanthin can help to dissipate excessive light energy to heat but also fulfils other roles, such as being an antioxidant against reactive oxygen species (ROS; García‐Plazaola et al., 2012; Roach et al., 2015). The xanthophyll (VAZ) cycle was also shown to be inducible by freezing stress in several fern species, despite darkness (Fernández‐Marín et al., 2021a), which is in line with a protective function of zeaxanthin under multiple stresses (Fernández‐Marín et al., 2021b). While most studies focus on vascular land plants, less is known about the impact of freezing events and cold acclimation in aquatic and terrestrial algal groups. This represents a great gap in knowledge, not only about the persistence of complex ecosystems in the aspect of climate change but also about the process of terrestrialization, as land plants (Embryophyta) evolved from streptophyte algae.

Around 720 and 635 million years ago, most terrestrial habitats were covered in snow or ice (Žárský et al., 2022). It is during this time period (Cryogenian) that the last common ancestor of Embryophyta and its closest streptophyte algal relatives (Zygnematophyceae) arose (Žárský et al., 2022). This group, termed Anydrophyta, was faced with the extreme environments of Snowball Earth, and adaptations to such cold conditions were crucial. It is proposed that the ancestral cellular adaptations to glacial habitats were exapted in streptophyte algae and a key factor for the process of terrestrialization (Žárský et al., 2022). Therefore, studying the effects of cold and freezing stress in streptophyte algae, particularly in Zygnematophyceae, is crucial to expand our understanding of early land plant evolution.

Streptophyte algae, grouped into the KCM‐grade (Klebsormidiophyceae, Chlorokybophyceae and Mesostigmatophyceae) and ZCC‐grade (Zygnematophyceae, Coleochaetophyceae and Charophyceae; de Vries et al., 2016), are essential parts of soil and freshwater aquatic ecosystems, with some still inhabiting glacial environments today (Žárský et al., 2022). In this aspect, studies conducted on *Klebsormidium* (Klebsormidiophyceae) have demonstrated dynamic adaptation strategies and the ability to withstand freezing and desiccation injuries (Elster et al., 2008; Nagao et al., 2008; Steiner et al., 2020). While *Klebsormidium* is a common part of biological soil crusts in extreme habitats, members of the ZCC‐grade also possess a wide geographical distribution. In particular, Zygnematophyceae algae inhabit extremely cold environments like the Arctic (Sheath et al., 1996; Kim et al., 2008, 2011; Pichrtová et al., 2016) and Antarctic (Hawes, 1989; Davey, 1991; Skácelová et al., 2013) and have been identified as major primary producers in polar hydro‐terrestrial habitats (Elster, 2002; Pichrtová et al., 2018; Williamson et al., 2019). Their persistence, especially in cold environments, is highly relevant for the continuity and function of these ecosystems, but their adaptations to freezing stress remain underexplored.

Macroscopically, Zygnematophyceae often appear as slimy filamentous mats and although not discernible as such due to their simple body plan, they have been established as immediate sister lineage to land plants (de Vries & Archibald, 2018; Leebens‐Mack et al., 2019). Their occurrence in often extreme and semi‐terrestrial habitats also exposes them to increased levels of other abiotic stresses like high UV radiation, drastic temperature shifts as well as desiccation stress. To deal with these challenges, Zygnematophyceae have evolved a variety of different adaptation strategies and avoidance mechanisms. Multiple detailed reviews on the abiotic stress tolerance of streptophyte green algae have been conducted over the past decade (Holzinger & Karsten, 2013; Holzinger & Pichrtová, 2016; Becker et al., 2020; Permann et al., 2022a). Just recently, a comprehensive study on the morphological, photophysiological, and transcriptomic response of two Zygnematophyceae to desiccation stress was also performed (Rieseberg et al., 2023). Briefly, vegetative filaments have been shown to accumulate cellular storage/ protective compounds and to increase their cell wall thickness for protection (Herburger & Holzinger, 2015; Pichrtová et al., 2016; Holzinger et al., 2018; Herburger et al., 2019). Asexually formed resting stages are also often formed during unfavourable environmental conditions, enabling the survival of the population (Kadlubowska, 1984; Fuller, 2013). Additionally, Zygnematophyceae are characterized by their special form of sexual reproduction via conjugation. Conjugation leads to the formation of zygospores, which exhibit a multi‐layered cell wall with a highly complex ultrastructure and compounds resistant to abiotic stresses, not found in vegetative cells (Poulícková et al., 2007; Permann et al., 2021a, b, 2022b).

While many studies focused on the stress tolerance of Zygnematophyceae against UV radiation and desiccation stress, less is known about their adaptation strategies to freezing events. Trumhová et al. (2019) established a high frost hardiness in mature *Zygnema* cells, termed pre‐akinetes, which are formed at the end of the growing season, exhibiting an LT_50_ value of −26.2°C. These cells could occasionally even survive treatments down to −70°C. Pre‐akinetes have furthermore been demonstrated to recover from freeze–thaw cycles (Pichrtová et al., 2016). Pronounced seasonality has been reported in many Zygnematophyceae (Hawes, 1988; Pichrtová et al., 2016; Trumhová et al., 2023) and it is speculated that while young dividing cells are dominant at the beginning of the vegetation period, resistant spores or resting stages are vital to survive freezing events (Hawes, 1988, 1990; Pichrtová et al., 2016, 2018; Trumhová et al., 2019, 2023; Arc et al., 2020). Nevertheless, young vegetative cells have also been demonstrated to tolerate freezing events down to −2/−4°C (Hawes, 1990; Trumhová et al., 2019; respectively). Additionally, the ability of cold acclimation leading to a significantly enhanced frost hardiness has been shown in *Klebsormidium flaccidum* (Nagao et al., 2008). Some streptophyte algae, in contrast, exhibit an extremely high sensitivity to freezing events, unable to survive ice formation. To survive the winter season, such algae rely on the avoidance of subzero temperatures and/or freezing events by snow or ice coverage, which effectively thermally insulates waterbodies (Hawes, 1990; Steiner et al., 2021; Trumhová et al., 2023). *Micrasterias denticulata* (Zygnematophyceae) cultures were shown to be ice susceptible and incapable of frost hardening but survive the winter season by migrating into deeper layers of peat bog pools, which are then covered by insulating snow (Steiner et al., 2021), a strategy most likely also applied by many other streptophyte algae.

Overall, Zygnematophyceae are essential in many semi‐terrestrial ecosystems and stream waters, including polar and high elevation habitats, which exhibit pronounced seasonal differences and long periods of frost (Marchland, 2014; Trumhová et al., 2023). As late spring freezing events are predicted to increase as a result of climate change (Ault et al., 2015; Liu et al., 2018; Zohner et al., 2020; Chamberlain & Wolkovich, 2021), studies concerning their frost hardiness and/or avoidance strategies are highly relevant. Such data also plays a key role in understanding the early land plant evolution as the split of Zygnematophyceae and Embryophyta is suggested to have taken place during the late Cryogenian (Žárský et al., 2022), a geological era characterized by extended cold glacial environments.

Concerning the effects of temperature extremes on streptophyte algae, most studies have been conducted on the genera *Micrasterias, Klebsormidium*, and *Zygnema*. While *Mougeotia* has served for a long time as a cell biological research object to study, e.g. chloroplast movements (e.g. Wagner et al., 1972; Wagner & Klein, 1981) and recently the effects of severe heat stress or submergence on physiological responses and transcriptomic changes were studied in this genus (de Vries et al., 2020; Fürst‐Jansen et al., 2021), to the best of our knowledge, no data on cooling or freezing effects are available.

The present study investigated the frost hardiness of two *Mougeotia* species from different habitats, i.e. *M. disjuncta* from a shallow water body in Kühtai, Austria, located at 2020 m a.s.l. and *M. scalaris* from a pond in Göttingen, Germany, located at 700 m a.s.l. We hypothesize that (1) species exposed to freezing temperatures in their natural habitat, such as the alpine *Mougeotia* species, exhibit higher frost hardiness than lowland species that avoid frost exposure or ice encasement during the winter season, (2) frost hardiness increases after cold acclimation and (3) freezing events will lead to increased levels of reactive oxygen species (ROS) and VAZ cycle activation, even in darkness. Characterizations based on phylogenetic (*rbc*L sequence), morphological, ultrastructural, and physiological data were performed. The two species were exposed to subzero temperatures and/or induced freezing events and analyzed for viability (measured by the effective quantum yield of photosystem II (Φ_PSII_)), morphology (cryo‐microscopy), ultrastructure (transmission electron microscopy (TEM)), hydrogen peroxide (H_2_O_2_) production, as well as photosynthetic pigment content. Additionally, cultures were cold acclimated for 3 weeks at +4°C prior to the freezing experiment to investigate its effect on their frost hardiness. The presented data will provide insights into the adaptation capabilities as well as the freezing stress management of Zygnematophyceae and, subsequently, their long‐term persistence in habitats increasingly exposed to untimely cold snaps.

## 2 MATERIALS AND METHODS

### 2.1 Algal species, habitat and culture conditions

The present study investigates two *Mougeotia* species: 1) *Mougeotia disjuncta* isolated from Kühtai (Tyrol, Austria; 47°21′76”N, 11°03′77″E; 2020 m a.s.l.) in 2020 and deposited in the Culture Collection of Algae at Göttingen (SAG; SAG 2658). This species was isolated from a single germinating zygospore, and the identification was based on zygospore and conjugation characteristics (for details, see Permann et al., 2021b). The habitat was characterized as slow running rivulets or shallow pools of water (Permann et al., 2021b). The climate in Kühtai is characterized as Dfb (winter‐wet‐cold with warm summer) according to the Köppen and Geiger (1936–1939) classification. The average annual temperature is 0.9°C, and the annual precipitation is 1653 mm (Climate, 2023). 2) *Mougeotia scalaris* (SAG 164.80) obtained from the SAG, which was originally isolated from a pond at the Botanical Garden at the University of Göttingen (Göttingen, Germany; 51°11′34.9”N, 6°48′23.9″E; 700 m a.s.l.) in 1975. The climate in Göttingen is characterized as Cfb (humid‐temperate with warm summer) according to the Köppen and Geiger (1936–1939) classification. The annual mean temperature is 9.2°C, and the annual precipitation is 804 mm (Climate, 2023). Both species were cultivated on Bold's Basal Medium solidified in Petri dishes with 1.5% agar. Plates were exposed to a 16/8 h light/dark cycle with a photon flux density of about 60 μmol photons m^−2^ s^−1^ and 20/15°C, respectively. For the cold acclimation experiment, cultures were cultivated at +4°C at a 12/12 h light/dark cycle for 3 weeks, a standard cold stress treatment for higher plants, like *Arabidopsis* (e.g. Zuther et al., 2012; Leuendorf et al., 2020). All measurements and experiments were performed on 6 to 8‐week‐old cultures.

### 2.2 DNA extraction and phylogenetic analysis

Cells of the two *Mougeotia* species were each collected and rinsed in sterile BBM and then drained to remove most of the liquid medium. Extraction of genomic DNA of *M. scalaris* was performed using the ZymoBiomics DNA prep kit (ZymoResearch). DNA extraction of *M. disjuncta* used CTAB extraction buffer (Doyle & Doyle, 1987) but without beta‐mercaptoethanol, followed by chloroform:isoamyl alcohol (24:1 v/v) extraction and centrifugation. DNA in the aqueous layer was precipitated with cold isopropanol and then resuspended in TE buffer. PCR amplification of the *rbc*L region was performed using primers M28 plus M1161r or M1338r (McManus & Lewis, 2011) and GoTaq Green Master Mix (Promega). The resulting products were sequenced commercially (Eurofins) using those same primers. The resulting sequencing reads from each taxon were trimmed manually to remove ambiguous bases at the ends and then assembled into contigs in Geneious Prime 2023.1.2 (Biomatters, Inc.). Each consensus sequence was used for a BLAST analysis, and the nearest published sequences were used to prepare a single alignment. Phylogenetic analysis included Maximum Likelihood (ML) and Bayesian Inference. The GTR + I + gamma model was indicated in Modeltest in PAUP* 4.0a build 169 (Swofford, 2003) under AIC, which was used for an ML analysis followed by bootstrapping (1000 replicates). Bayesian Inference was done using the MrBayes Plugin Build 2.2.4 (Huelsenbeck & Ronquist, 2001; Ronquist & Huelsenbeck, 2003) in Geneious under the GTR + I + gamma model. A majority rule consensus tree was built after two runs of 106 generations, after discarding the initial 25% of trees per run as burnin.

### 2.3 Light‐, fluorescence‐, and confocal laser scanning microscopy

Light microscopical (LM) images of the filaments were taken on a Zeiss Axiovert 200 M light microscope (Carl Zeiss AG), equipped with an Axiocam HRc camera (Carl Zeiss AG) and a Zeiss Axiovision software. Cell measurements (n = 35) were then taken via ImageJ/ Fiji (Schindelin et al., 2012). Confocal laser scanning microscopy (CLSM) was performed with a Zeiss Pascal system under the control of Zen 2009 software, excitation was generated with an argon laser (488 nm), and emission was collected with a long pass filter (505 nm), and the chloroplast autofluorescence was false coloured red. Z‐stacks were generated from a series of 19 and 30 images for *M. disjuncta* and *M. scalaris*, respectively, at 1 μm distance and projected in the z‐axis. After the freezing experiments (described below), light, as well as fluorescence micrographs, were taken, with the chlorophyll (Chl) autofluorescence being visualized with a Zeiss Filter Set 09 (Excitation: band pass (BP) 450**–**490 nm and emission: long pass (LP) 515 nm). Images were processed using Adobe Photoshop Elements 11 (Adobe Inc., 2019).

### 2.4 Chemical fixation and high‐pressure freeze fixation

On untreated cells of both species, chemical fixation and high‐pressure freeze‐fixation (HPF) + freeze substitution (FS) were performed. Chemically fixation was performed according to Holzinger et al. (2009). In summary, cells were fixed in 2.5% glutaraldehyde (20 mM cacodylate buffer, pH 7) for 1.5 h and afterwards rinsed with 20 mM cacodylate buffer. They were then embedded in 3% agarose (distilled water) and post‐fixed in 1% OsO_4_ (20 mM cacodylate buffer) at 4°C overnight. Dehydration was performed using increasing concentrations of ethanol and propylene oxide and the samples embedded in modified Spurr's resin. For HPF and FS, the protocol of Aichinger and Lütz‐Meindl (2005) was applied. Briefly, samples were fixed with a LEICA EMPACT high‐pressure freezer and freeze substituted in a Leica EM AFS FS apparatus (Leica Microsystems GmbH) in 2% OsO_4_ and 0.05% uranyl acetate in acetone. Samples were exposed to −80°C for 60 h, followed by −30°C (warming rate 10°C h^−1^) for 4 h and finally 20°C (warming rate 2.5°C h^−1^). Afterwards, they were embedded in an Agar low viscosity resin kit (Agar Scientific).

### 2.5 Transmission electron microscopy

For Transmission electron microscopy, chemically fixated or HPF/FS samples were sectioned with a Reichert Ultracut (Leica Microsystems) and the ultrathin sections (~60–90 nm) stained with 2% uranyl acetate and Reynold's lead citrate. Images were taken on a Zeiss Libra 120 transmission electron microscope (Carl Zeiss AG) at 80 kV, which was equipped with a TRS 2 k SSCCD camera and operated by ImageSP software (Albert Tröndle Restlichtverstärker Systeme).

### 2.6 Osmotic stress treatment

Fresh algal material of both *Mougeotia* species was incubated for 1 h in 300, 400, 500, 600, 700, 800, 900 and 1000 mM D‐sorbitol solutions, and images captured with a Zeiss Axiovert 200 M light microscope (described above). A minimum of 100 cells were used per solution and species for further analysis.

### 2.7 Oxygen measurements, rapid light curves and non‐photochemical quenching

Characterization of the photosynthetic performance was done by measuring oxygen evolution, relative electron transport rates (rETRs) and non‐photochemical quenching (NPQ). Photosynthetic oxygen production (P_gross_) and dark respiration (R_d_) were measured as described in Karsten and Holzinger (2012) and Pierangelini et al. (2019) and used for determining the net oxygen production (P_net_). In summary, algal filaments were cut into smaller fractions and resuspended in 3 mL distilled water and 60 μL inorganic carbon solution (0.1 M Na_2_CO_3_ and Na_2_HCO_3_, 1:19). The suspension was placed into a thermostatic acrylic chamber (type DW1, Hansatech Instruments) and the oxygen production measured with a Presens Fibox 3 oxygen optode (Presens). The algae were exposed to increasing temperatures (5**–**40°C) with 5°C step increments. For every step, the cells were dark incubated for 30 min, with the last 10 min used for R_d_ calculation, and afterwards exposed to 200 μmol photons m^−2^ s^−1^ for 10 min, with the last 5 min used for P_gross_ calculation. The Chl *a* content was determined by DMF extraction, following the protocol of Pierangelini et al. (2017). Relative values were calculated according to the following formula: $\frac{\text{oxygen absolute}\times \text{volume}\times 60}{\mathrm{chl}\;a\;\text{absolute}\times \text{measuring periode}}$. For rapid light curve (RLC) determination, based on rETRs, cells were exposed to increasing light intensities (every 30 s) of 0 to 2013 μmol photons m^−2^ s^−1^ using a PAM 2500 fluorimeter (Heinz Walz). NPQ curves were obtained as described in Pierangelini et al. (2017). Briefly, cells were exposed to five saturating light pulses (300 ms) during exposure to actinic light (618 μmol photons m^−2^ s^−1^), which was followed by a dark recovery time to monitor the NPQ relaxation phase. All measurements were performed in triplicates.

### 2.8 Determination of frost hardiness

For analysing the level of frost hardiness, 20–22 mg of fresh algal material was harvested and put into 2 mL Eppendorf tubes filled with 1 mL of distilled water to avoid desiccation stress. The cells were exposed to different freezing temperatures (−1, −2, −3, −5, −7, −9°C). Exposure was in commercial freezers (GT series, Liebherr), which were customised to be fully temperature controllable, as described in Neuner et al. (2020). To investigate the effects of ice formation *per se*, −1°C samples were kept free of ice, whereas at −2°C, the samples were either ice nucleated or kept ice‐free. From −3°C downwards samples were always ice nucleated. Ice nucleation was artificially triggered via a precooled preparation needle. The temperatures were monitored via thermocouples directly attached to the outer surface of the Eppendorf tubes. Cooling and warming rates were set at 3 K h^−1^, resembling natural conditions, and the cells were kept at the target temperature for 10 h. This setup was repeated with cold acclimated cultures (as described above). After experimental freezing, the samples were returned to the culture conditions as described above.

For determining the viability of the samples, the effective quantum yield of photosystem II (PS II; Φ_PSII_) was measured using a PAM 2500 fluorometer (Heinz Walz GmbH) 1, 3, 6, and 24 h after the freezing experiment. For the cold acclimation experiment, the samples were returned to the culture conditions prior to the freezing experiments (+4°C) and Φ_PSII_ measured at the same time intervals. In addition, their recovery in standard culture conditions was monitored. For this, the samples were returned to their initial temperature and light regime (see above) and Φ_PSII_ was measured 25, 27, 30, and 50 h after the freezing experiment. The viability level was then calculated as a percentage of the initial Φ_PSII_, which was measured before the freezing experiments. All measurements were performed in triplicates.

To compare degrees of frost hardiness, lethal temperatures at 50% frost damage (LT_50_) were calculated. For this, a logistic function was fitted to the Φ_PSII_ values (24 h after the treatment), which determined the temperature at which 50% of tested plant material is considered as damaged; for the detailed protocol, see Stegner et al. (2020). To the fitted curves, we also calculated the 95% confidence intervals. The frost hardiness was only considered to be significantly different if the different species/approaches did not overlap in their confidence intervals.

### 2.9 Freeze experiments for ultrastructure, pigment and H_2_O_2_ analyses

Sample preparation for TEM, pigment and H_2_O_2_ analysis followed the four experimental setups at a target temperature of −2°C: (1) samples harvested after 5 h at target temperature (V), (2) samples harvested after full cycle, including 10 h at target temperature (X), (3) samples harvested in a frozen state after 5 h at target temperature with ice nucleation (V_F_), and (4) samples harvested after full cycle, including 10 h at target temperature with ice nucleation and subsequent thawing (X_F_). Samples were also harvested before the experiment as control (C). All experiments were performed in triplicates. For analysing the ultrastructural changes after the treatments X and X_F_, the samples were chemically fixed and viewed via TEM, as described above.

### 2.10 Pigment analysis

Pigments were analysed via high pressure liquid chromatography (HPLC) before the experiments (C) and after the treatments V, X, V_F_ and X_F_ (described above). For this, 2 mg of lyophilised algal biomass together with two 5 mm agate beads was placed in 2 mL Eppendorf tubes and disrupted by a TissueLyser (TissueLyser II; Qiagen) at 30 Hz for 3 min in pre‐cooled (−20°C) racks. Afterwards, 700 μL of MeOH were added, and the Tissuelyser step was repeated. Samples were centrifuged at 26.000 *g* and 4°C for 45 min (Sigma 3‐18 K Centrifuge/ SciQuip Ltd.). Pigments in 10 μL of supernatant were separated on a LiChrospher 100 RP‐18 column (125 × 4 mm, 5 μm), using a gradient between mobile phases ACN/ MeOH (74:6) and MeOH/ hexane (5:1), using an Agilent 1100 Series HPLC connected to a G1315B DAD diode array detector (Agilent Technologies). Pigments were quantified by absorption at 440 nm using external standards.

### 2.11 Determination of H_2_O_2_ concentration

After the two experimental setups X and X_F_ (described above) the incubation water, in which the algae were treated, was collected for H_2_O_2_ measurements. The diluted sample water (1:3 in *double‐distilled H*_*2*_ *O*) was combined with a reaction mix consisting of 1% 2 mM Amplex™ Red Reagent substrate, 4% 20 U/mL horseradish peroxidase and buffer (0.5 M Tris–HCl, pH 7.5), according to manufacturer's protocol (Invitrogen™, Thermo Fisher Scientific Inc.). Fluorescence measurements (Ex: 570 nm, Em: 585 nm) of H_2_O_2_‐dependent resorufin product were conducted using an Agilent multi‐mode microplate reader Synergy™ HTX (Agilent Technologies) controlled by Agilent BioTek Gen 6 software. Concentrations of H_2_O_2_ were calculated against an external standard.

### 2.12 Cryo microscopy

Cryo microscopical images were taken on a Leica DM1000 light microscope (Leica Microsystems GmbH), equipped with a Leica EC4 camera and Leica software LAS EZ 3.0. The microscope was placed inside a cooling compartment of a temperature‐controlled freezer (GT series, Liebherr; see above), which was covered by a customized acrylic glass lid with integrated thermally insulated gloves, enabling the operation of the microscope without affecting the temperature inside the chamber. Algal filaments were placed on a microscope slide, and the temperature under the coverslip was monitored by thermocouples. To prevent desiccation stress and inducing ice nucleation, cotton fibres, which protruded from the cover slide, were added to the sample. The cooling rate was set at −3 K h^−1^ and images were taken before, during and after induction of ice nucleation.

### 2.13 Statistical evaluation

The statistical evaluation of the data was performed via two‐sample *t*‐tests or multifactorial ANOVA analysis, followed by Tukey's post hoc‐test using RStudio 4.1 (R Core Team, 2021). Model assumptions were tested using Levene's test and residual diagnostic. All calculated values are available in the supplementary material of this article (**Tables** **S1‐S5**).

## 3 RESULTS

### 3.1 Phylogenetical position of the focal *Mougeotia* strains

**FIGURE 1:**
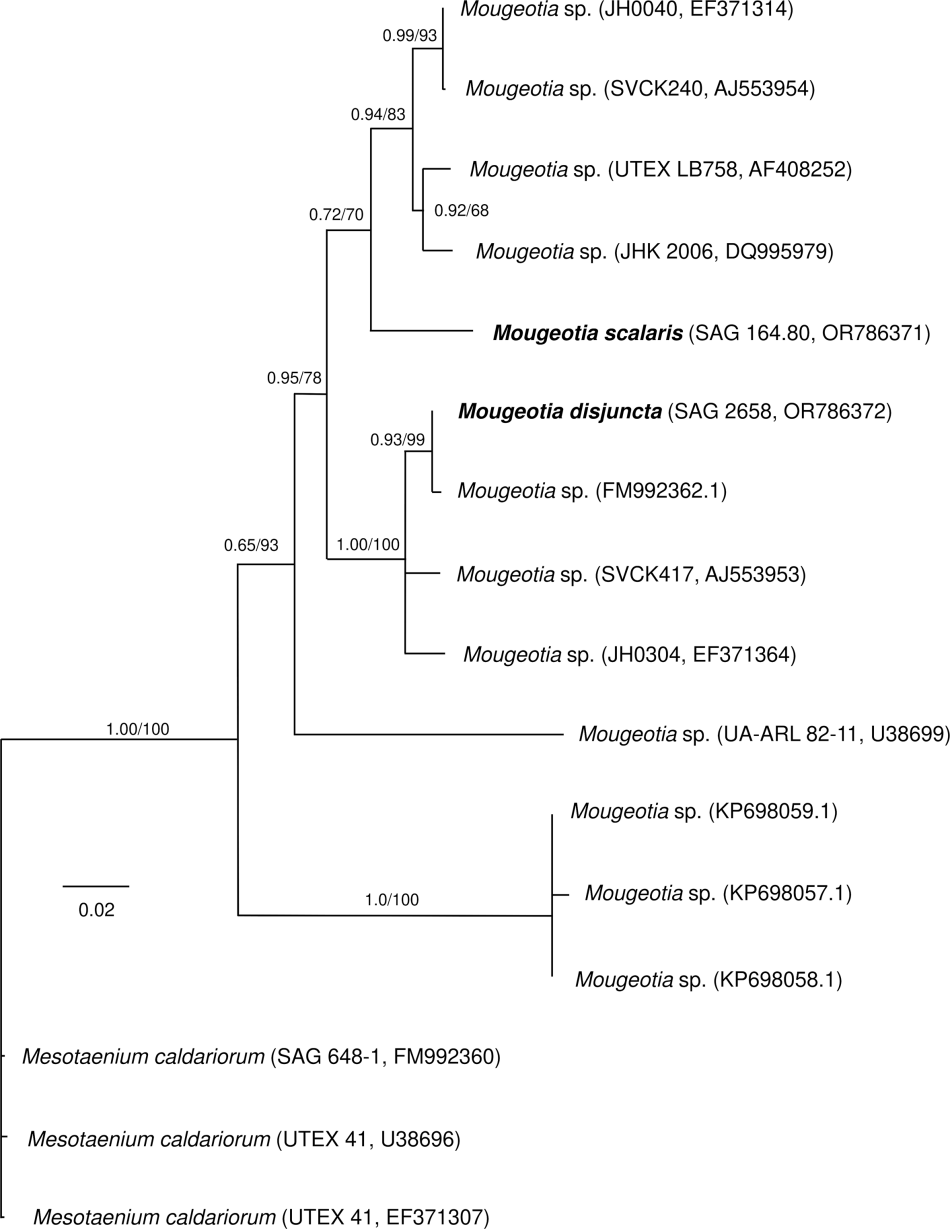
Phylogenetic placement of focal strains *Mougeotia disjuncta* (SAG 2658) and *Mougeotia scalaris* (SAG 164.80) based on *rbc*L sequences, oriented with three sequences of *Mesotaenium* as the outgroup. The tree was obtained using maximum likelihood (ML) and nodes are labelled with Bayesian posterior probabilities/ML bootstrap support values. Unlabeled nodes were absent in the Bayesian consensus tree or received bootstrap values below 51. Taxon labels include the strain designation and the NCBI/ EMBL accession number. The scale bar indicates the expected number of substitutions per site.

Partial *rbc*L sequences were obtained for the two species, with a length of 1041 base pairs (bp) for *M. disjuncta* (sequence deposited in NCBI GeneBank under accession number: OR786372) and 1222 bp for *M. scalaris* (sequence deposited in NCBI GeneBank under accession number: OR786371). Phylogenetic analysis of these two, plus eleven published sequences of other *Mougeotia* accessions, placed the two investigated strains in different clades (Figure 1). *Mougeotia disjuncta* was most closely related to *Mougeotia* sp. (FM992362.1), while *M. scalaris* was positioned in a sister clade, which included four other strains of *Mougeotia* spp.

### 3.2 Morphological and ultrastructural characterization

The investigated *Mougeotia* species showed clear morphological differences upon LM and CLSM analyses. *Mougeotia disjuncta* exhibited an average cell length of 52.9 ± 7.6 μm, a cell width of 15.3 ± 1.3 μm and a length:width ratio of 3.5 ± 0.6. The vegetative filaments of *M. scalaris*, in contrast, were larger in size, with an average cell length of 155.3 ± 31.4 μm, width of 27.0 ± 3.2 μm and a length:width ratio of 5.8 ± 1.4. All aforementioned cell measurements differed significantly (*p* < 0.001) between the two species.

**FIGURE 2:**
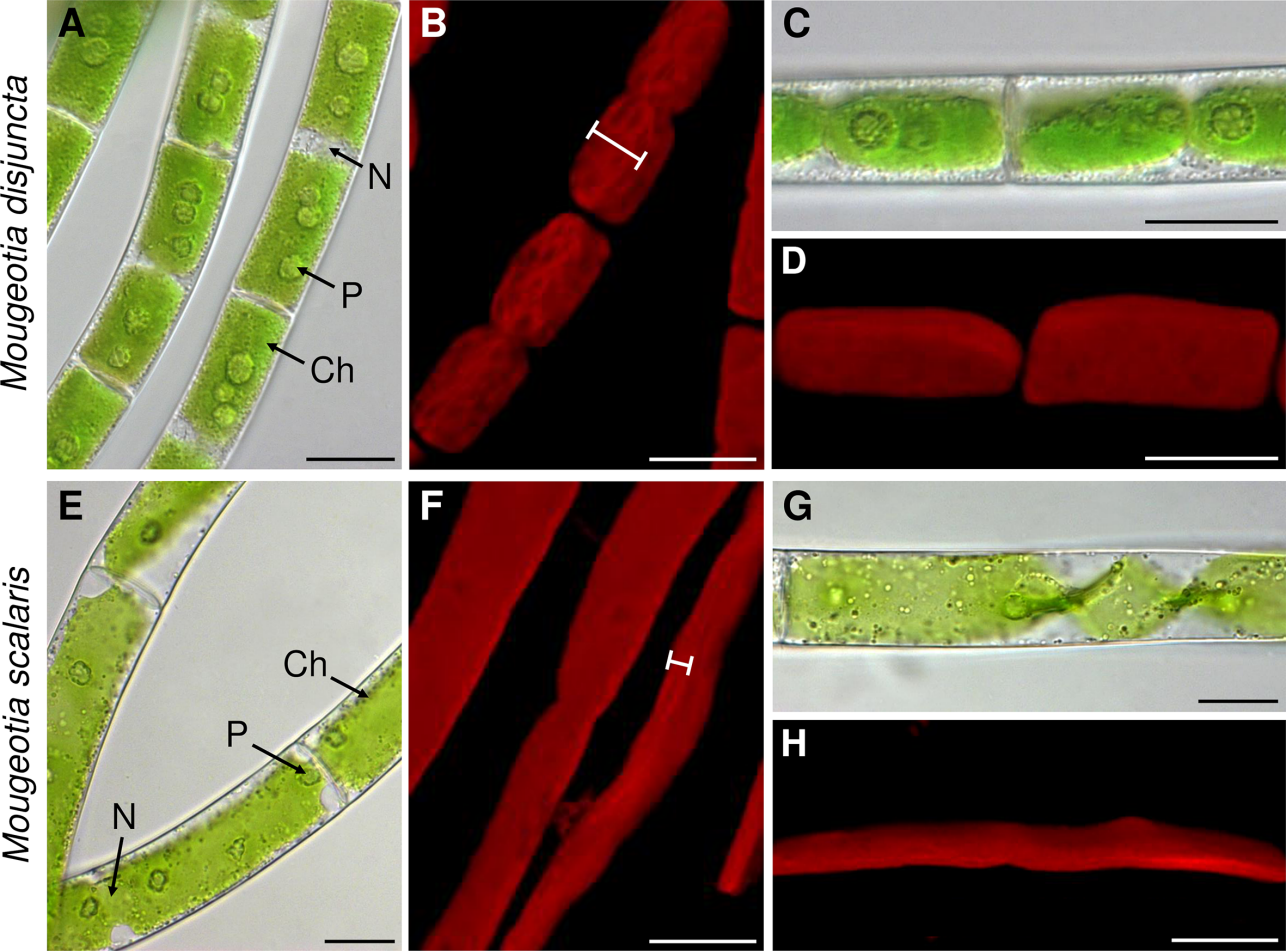
Morphological characterization of vegetative *Mougeotia* filaments by light and confocal laser scanning microscopy. **(A–D*)*** *Mougeotia disjuncta*; **(E–H)** *Mougeotia scalaris*; **(A, C, E, G)** light micrographs; **(B, D, F, H)** confocal laser scanning micrographs (chlorophyll autofluorescence (red); z‐stack projections); **(A, B, E, F)** vegetative filaments (bars indicating chloroplast diameter) with **(C, D, G, H)** detail view of the chloroplast shape, correspondingly. Abbreviations: Ch, chloroplast; N, nucleus; P, pyrenoid. Scale bars = 20 μm.

**FIGURE 3:**
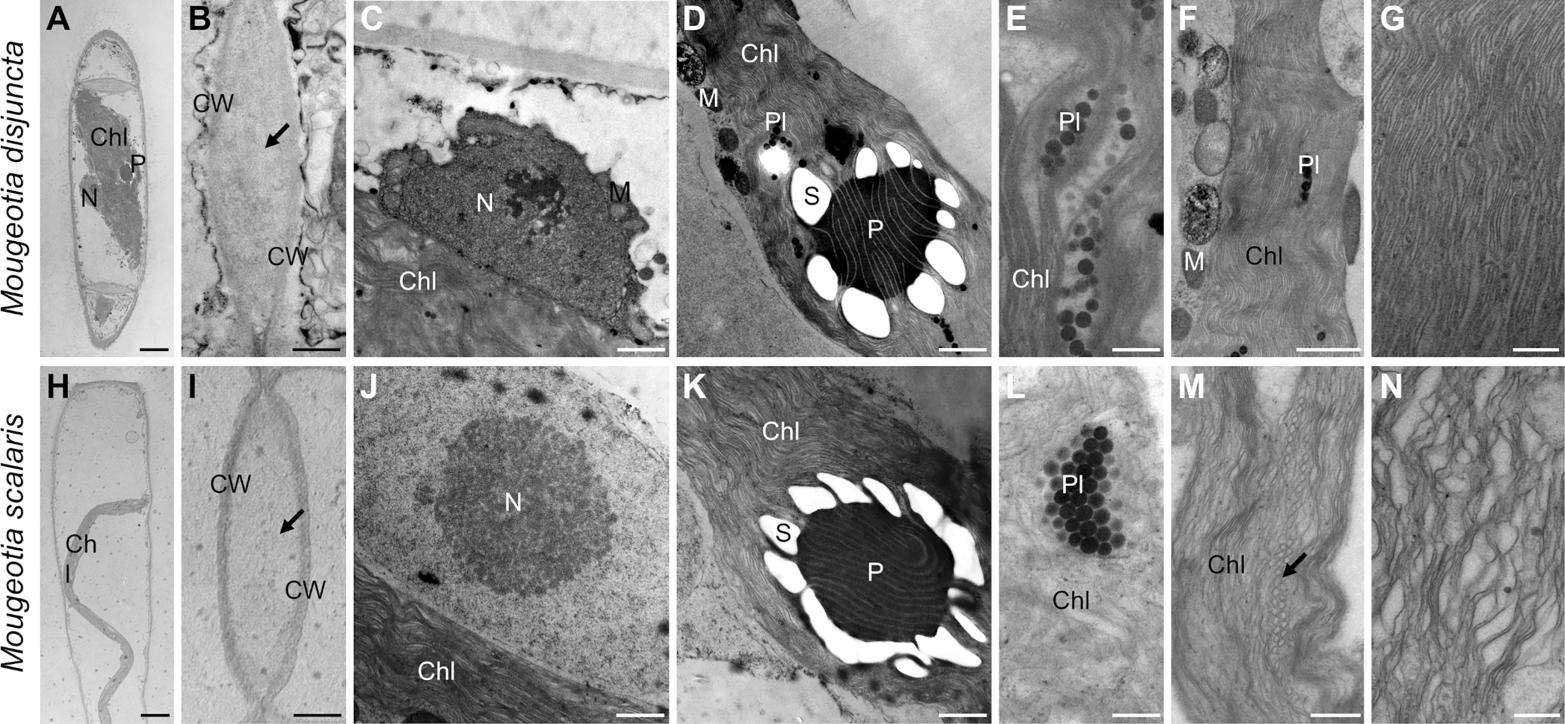
Ultrastructural characterization by transmission electron microscopy of **(A–G)** *Mougeotia disjuncta* and **(H–N)** *Mougeotia scalaris*. (**A–C, E, G, H, I, L–N**) chemical fixation, **(D, F, J, K)** high‐pressure freeze‐fixation and freeze substitution **(A, H),** cell overview depicting difference in chloroplast dimensions between the two species; **(B, I)** cell–cell conjunction with loose polysaccharidic material in between (arrows); **(C, J)** detail view of nucleus; **(D, K)** detail view of pyrenoid surrounded by starch grains and thylakoid membranes; **(E, L)** detail view of accumulated plastoglobules embedded in the thylakoid membranes of the chloroplast; **(F)** chloroplast with adjacent mitochondria; **(G)** dense arrangement of the thylakoid membranes; **(M)** detail view of thylakoid superstructure showing gyroid cubic membranes (arrow); **(N)** loose arrangement of the thylakoid membranes. Abbreviations: Chl, chloroplast; CW, cell wall; M, mitochondria; N, nucleus; P, pyrenoid; Pl, plastoglobules; S, starch grain. Scale bars: (A, H) 5 μm; (B**–**D, F, J, K) 1 μm; (E, G, L**–**N) 500 nm; (I) 2.5 μm.

Another striking difference was found in the chloroplast shape and vacuole size. *Mougeotia disjuncta* showed thick yet still plate‐like and not parietal chloroplasts, which occupied almost the entire cell lumen (Figure 2A, C). The chloroplasts of *M. scalaris*, in contrast, were thinner and surrounded by large vacuoles (Figure 2E, G). The difference in chloroplast shape was further demonstrated by CLSM (Figure 2B, D, F, H) and TEM (Figure 3A, H). The ultrastructural investigations also revealed a loose polysaccharidic material located between the cell walls of two adjacent cells, which was present in both species (Figure 3B, I). Typical cell organelles like the nucleus, pyrenoids and chloroplasts with numerous plastoglobules were also equally observed (Figure 3C–F; J–L). Differences between the two *Mougeotia* species, however, were found in the structure and density of the thylakoid membranes. In *M. scalaris*, occasionally distinctive gyroid cubic membranes were formed, giving a moniliform appearance (Figure 3M). This species also exhibited a loose arrangement of thylakoid membranes while they were densely packed in *M. disjuncta* (Figure 3G, N).

### 3.3 Osmotic values and pigment content

**FIGURE 4:**
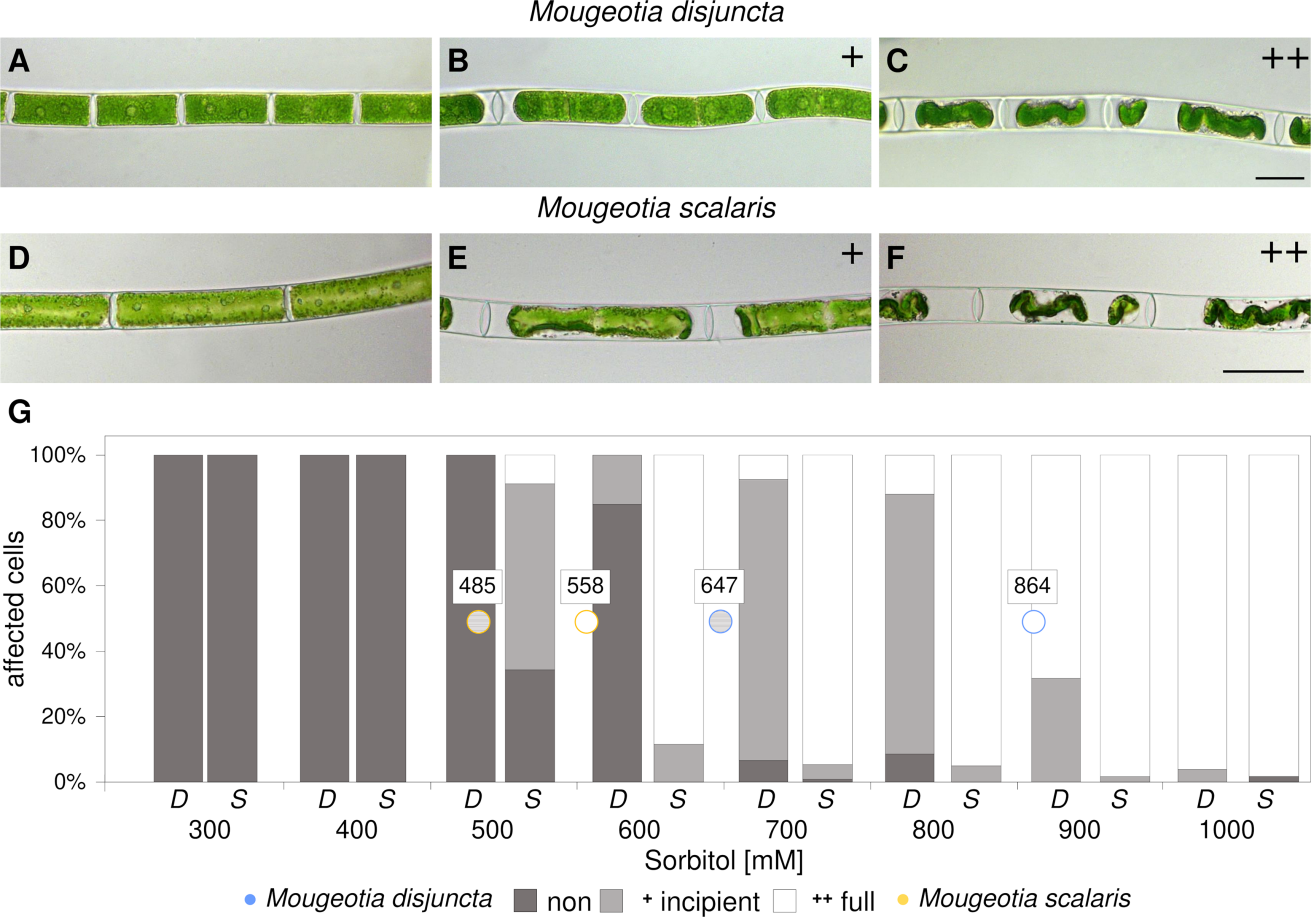
*Mougeotia disjuncta* **(A*–*C)** and *Mougeotia scalaris* **(D*–*F)** filaments exposed to increasing levels of sorbitol (300 mM *–* 1000 mM). **(A*–*F)** light microscopical images representing different levels of plasmolysis (levels indicated by: + incipient; ++ full); **(G)** percentages of non‐, incipient and fully plasmolyzed cells are shown as bar chart. Calculated sorbitol concentrations of 50% incipient (grey) or full (white) plasmolysis are indicated by blue (*M. disjuncta*) and yellow (*M. scalaris*) circles. Abbreviations: D, *Mougeotia disjuncta*; S, *Mougeotia scalaris*. Scale bars: (A ***–*** C) 20 μm; (D ***–*** F) 50 μm.

Investigations concerning the osmotic potential and effects of plasmolysis also depicted a disparity between the two species. For *M. disjuncta*, the concentration, where 50% of the cells were at least incipiently plasmolysed, was calculated at 647 mM, while this point was reached at already 485 mM for *M. scalaris* (Figure 4A–G). Full plasmolysis of 50% of the observed cells was calculated to occur at 864 mM in *M. disjuncta* and 558 mM in *M. scalaris* (Figure 4A–G). In the course of freezing experiments (described below), additional analysis of the pigment content of the two species were conducted. *Mougeotia scalaris* exhibited higher total amounts of xanthophyll cycle pigments (*p* < 0.001) and violaxanthin (*p* < 0.001), as well as lower levels of DEPS (*p* = 0.036). The lowland species furthermore showed higher levels of lutein (*p* = 0.020) as well as a higher Chl *a*: *b* ratio (*p* = 0.016), caused by significantly lower levels of Chl *b* (p = 0.030).

### 3.4 Physiological performance

**FIGURE 5:**
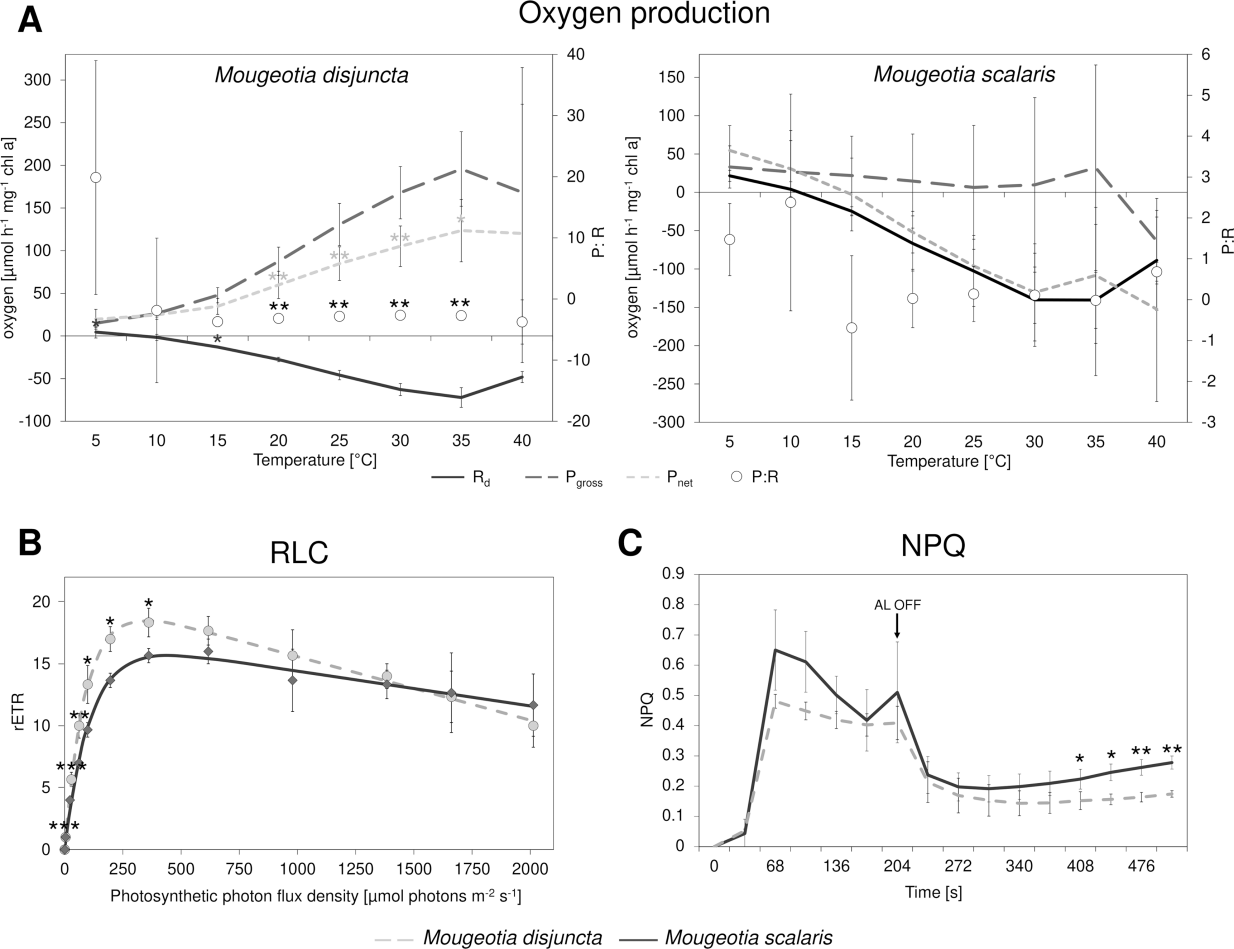
Photosynthetic performance of *Mougeotia disjuncta* and *Mougeotia scalaris*. **(A)** oxygen production (R_d_, P_gross_, P_net_, P:R ratio) for *M. disjuncta* (left) and *M. scalaris* (right). **(B)** relative electron transport rate (rETR) and **(C)** non‐photochemical quenching (NPQ) for *M. disjuncta* (dashed lines) and *M. scalaris* (solid lines). Mean values ± standard deviation. Significant differences between species are indicated by asterisks (p < 0.05*, p < 0.005**, p < 0.001***) and for oxygen production represented in the graph of *M. disjuncta* (left). Abbreviations: AL, actinic light; P_gross_, gross photosynthesis; P_net_, net photosynthesis; R_d_, dark respiration.

Comparisons of the physiological performance included measurements of oxygen production (R_d_, P_gross_, P_net_), rETRs as well as NPQ to gain an ecophysiological characterization of the investigated strains. While the two species did not significantly (*p* > 0.05) differ in P_gross_ and only at 5 and 15°C in R_d_ (*p* = 0.043 and 0.027, respectively), a significant difference was found in P_net_ at temperatures between 20–35°C (*p* < 0.05 Figure 5A). This difference was also reflected in the P:R ratio. Overall, *M. disjuncta* exhibited higher R_d_, P_gross_, and P_net_ values, while P_net_ in *M. scalaris* dropped below zero already at 15°C. The P:R ratio of both species showed the highest values at 5 and 10°C and stayed close to zero, with *M. disjuncta* exhibiting slightly lower values. RLCs, measured as rETRs in response to photon flux densities between 0–2013 μmol photons m^−2^ s^−1^, yielded differences only at PAR up to 361 μmol photons m^−2^ s^−1^ with decreasing significance levels (Figure 5B). Higher ETR_max_ values (18.57 ± 0.87) were reported in *M. disjuncta*. However, among the photosynthetic parameters of the RLCs, only the α value, which was higher in *M. disjuncta*, was found to differ significantly (*p* = 0.017; Table 1). Concerning the NPQ progression, overall values were higher in *M. scalaris*, but the species only differed at the end, after 408 s (Figure 5C) and not in NPQ_max_ or Φ_PSII_.

**TABLE 1:** Characteristic parameters of rapid light curves (α, positive slope at light‐limiting photon fluence rates; *l*_K_, initial value of light‐saturated photosynthesis), maximum non‐photochemical quenching (NPQ_max_), and yield of PSII (Φ_PSII_) of *Mougeotia disjuncta and Mougeotia scalaris*. Significant differences are indicated by asterisks (p < 0.05*, p < 0.005**, p < 0.001***)

Species	α *	*I*_k_	ETR_max_	Φ_PSII_	NPQ_max_
*Mougeotia disjuncta*	0.25 ± 0.03	75.20 ± 8.72	18.57 ± 0.87	0.55 ± 0.05	0.48 ± 0.02
*Mougeotia scalaris*	0.16 ± 0.02	102.43 ± 20.87	16.13 ± 1.30	0.57 ± 0.03	0.65 ± 0.13

### 3.5 Physiological performance and (ultrastructural) morphology after freezing experiments

**FIGURE 6:**
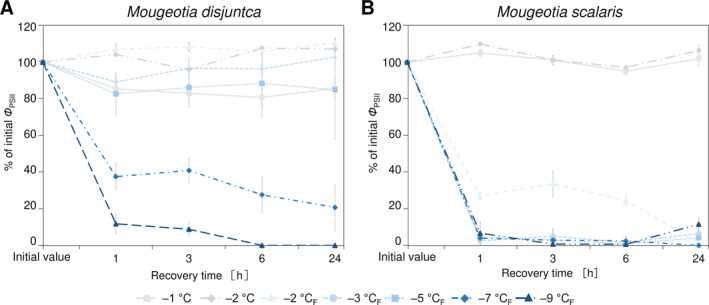
Changes in quantum yield of PSII (Φ_PSII_) in *Mougeotia* in response to exposure to subzero temperatures and/ or freezing; exposure time 10 h with cooling and warming rate of 3 K h^−*1*^. **(A)** *Mougeotia disjuncta*; **(B)** *Mougeotia scalaris*. (_F_) indicates treatments where ice nucleation was triggered. Mean values ± standard deviation.

**FIGURE 7:**
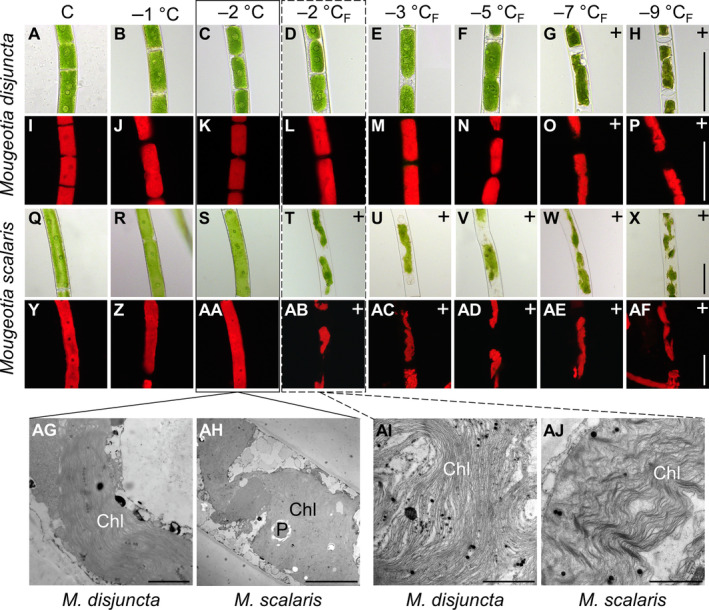
Morphological and ultrastructural analysis of **(A–P; AG, AI)** *Mougeotia disjuncta* and **(Q –AF, AH, AJ)** *Mougeotia scalaris* filaments 24 h after subzero temperature exposure and/ or induced freezing event; exposure time 10 h with cooling and warming rate of 3 K h^−1^. **(A–H, Q–X)** light microscopical images; **(I–P, Y–AF)** chlorophyll autofluorescence images; **(AG–AJ)** transmission electron microscopical images; **(AG, AH)** cells after exposure to **−**2°C without ice nucleation showing intact chloroplasts; **(AI, AJ)** cells after exposure to **−**2°C with induced ice nucleation showing changes to the thylakoid membrane structure. (_F_) indicates treatments where ice nucleation was triggered. (^+^) indicates visible chloroplast damage. Abbreviations: Chl, chloroplast; P, pyrenoid. Scale bars: (A**–**AF) 20 μm; (AG, AI, AJ) 2 μm; (AH) 10 μm.

The freezing experiments revealed clear differences in the frost hardiness between *M. disjuncta* and *M. scalaris*. *Mougeotia disjuncta* was able to recover from treatments down to –5°C with 84.9 ± 27.2% Φ_PSII_ of the initial value after 24 h (Figure 6A). While some photosynthetic activity was measured in cells exposed to −7°C (37.5 ± 7.3%) and − 9°C (11.8 ± 5.6%) 1 h after treatment, no recovery was observed after 24 h and both temperatures were considered lethal. The LT_50_ was calculated at −5.8°C (low confint −5.3°C/ high confint −6.3°C). *Mougeotia scalaris*, in contrast, showed ice susceptibility. Only samples exposed to −1 and − 2°C, where ice nucleation was omitted, survived (after 24 h: –2°C: 104.8 ± 0.2%; Figure 6B), which resulted in a calculated LT_50_ of −1.9°C (low confint −1.9°C/ high confint −2.0°C). 24 h after the freezing experiment, the viability of the filaments was also examined via light‐ and fluorescence microscopy. The morphological data gathered by LM support the measured Φ_PSII_, as damage to the chloroplasts was clearly visible in samples, exhibiting low Φ_PSII_ values (Figure 7A–AF). To further investigate the effects of ice formation, TEM analyses were performed on cells exposed to −2°C without ice formation (X) and − 2°C where ice nucleation had been triggered (X_F_). In accordance with the physiological performance and the LM investigations, no changes in ultrastructural features were observed after treatment X in either species (Fig. 7AG, AH). While no effects of treatment X_F_ were found in Φ_PSII_ or the LM chloroplast morphology in *M. disjuncta*, TEM images depicted a loosening of the thylakoid membrane arrangement (Fig. 7AI), suggesting ultrastructural alterations caused by ice formation. The ice susceptible *M. scalaris* also exhibited changes to the chloroplast ultrastructure in the form of rippled thylakoid membranes (Fig. 7AJ).

### 3.6 H_2_O_2_ measurements and pigment analysis

**FIGURE 8:**
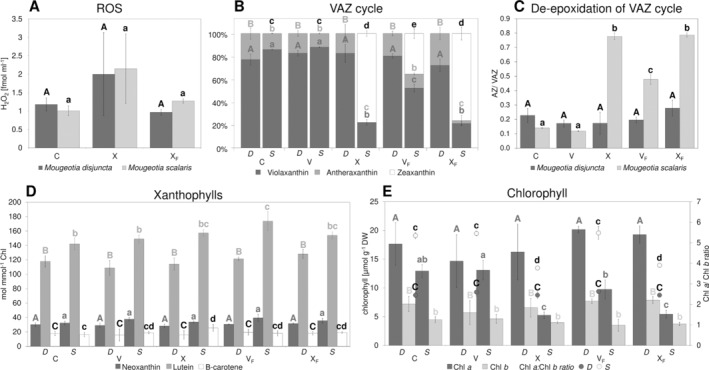
H_2_O_2_ production and pigment change in *Mougeotia* exposed to freezing stress at **−**2°C with and without triggered ice nucleation (C, control; V, exposure to 5 h at target temperature; X, exposure to full temperature cycle; V_F_, exposure to 5 h at target temperature + ice nucleation; X_F_, exposure to full temperature cycle + ice nucleation). **(A)** amount of released H_2_O_2_; **(B)** changes in VAZ cycle composition; **(C)** de‐epoxidation state of VAZ cycle (A + Z/ V + A + Z); **(D)** xanthophyll amount; **(E)** chlorophyll amount and ratio. Significant differences within species are indicated either by different capital letters (*M. disjuncta*) or different lowercase letters (*M. scalaris*), and by varying gray scale for each parameter (in B: Z, black; A, light grey; V, dark grey). Mean values ± standard deviation. Abbreviations: Chl, chlorophyll; *D*, *Mougeotia disjuncta*; *S*, *Mougeotia scalaris*; VAZ cycle, xantophyll cycle (V, violaxanthin; A, antherxanthin; Z, zeaxanthin).

The sample water was analysed for H_2_O_2_ after being subjected to a complete temperature cycle at −2°C, comparing responses without ice formation (X) with those where ice nucleation had been triggered (X_F_). While the mean amount of H_2_O_2_ was higher in X, no significant difference was found between the control samples (C) and both treatments (*p* > 0.05; Figure 8A). Additionally, changes in the pigment content were analysed, including samples harvested during the temperature cycle after 5 h at the target temperature (V/ V_F_; Figure 8B–E). The alpine *M. disjuncta* showed little changes in VAZ cycle composition and no formation of zeaxanthin. In *M. scalaris*, in contrast, the VAZ cycle was clearly activated in X, V_F_, and X_F_ (Figure 8B). Only samples of treatment V showed no significant (*p* > 0.05) difference to C. The changes included lower levels of violaxanthin and antheraxanthin and higher levels of zeaxanthin. This was also reflected in DEPS, with *M. disjuncta* remaining unaffected by the treatments and *M. scalaris* depicting elevated levels after X, V_F_ and X_F_ (Figure 8C). Interestingly, the amount of zeaxanthin and the DEPS showed no significant difference between X and X_F_ in the affected *M. scalaris*. The analysis of other xanthophylls (neoxanthin, lutein, and β‐carotene) revealed a significant difference in lutein between C and V_F_ in *M. scalaris* (*p* = 0.005). Regarding the chlorophyll content and ratio, X and X_F_ led to a decrease in Chl *a*: *b* ratio when compared to the control group in the lowland species *M. scalaris* (*p* < 0.001; Figure 8E).

### 3.7 Cold acclimation

**FIGURE 9:**
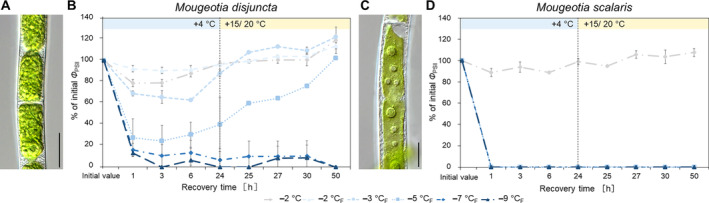
*Mougeotia* cultures after a 3‐week cold acclimation treatment at +4°C and effective quantum yield of PSII (Φ_PSII_) after freezing experiments. **(A, C)** *Mougeotia disjuncta* and **(C, D)** *Mougeotia scalaris*. **(A, B)** light micrographs; **(B, D)** Φ_PSII_ during recovery at +4°C (1**–**24 h) and at +15/ 20°C (24**–**50 h). (_F_) indicates that ice nucleation was triggered. Mean values ± standard deviation. Scale bars = 20 μm.

To investigate the cold acclimation ability in *Mougeotia*, additional experiments were conducted on cultures cultivated for 3 weeks at +4°C and checked for an increase in frost hardiness. After the cold treatment, visible changes in the form of accumulated storage compounds, especially in *M. disjuncta*, were observed (Figure 9A, C). While a significant difference in the initial Φ_PSII_ between standard cultivation and cold cultivation was found only in *M. scalaris* (*p* < 0.001), the change from cold conditions to standard culture conditions after the temperature cycle enhanced recovery in *M. disjuncta* and resulted in Φ_PSII_ higher than the initial values. However, no enhancement of frost hardiness was measured in either of the two species. In contrast, a significantly decreased LT_50_ value of −4.7°C (low confint −4.4°C/ high confint −5.0°C) was calculated for cold acclimated *M. disjuncta* (Figure 9B *)*. In the lowland species *M. scalaris*, the severity of ice nucleation was increased in comparison to untreated cultures, as Φ_PSII_ was not even detectable after the temperature cycle and no recovery was observed (Figure 9D).

### 3.8 Cryo microscopy

**FIGURE 10:**
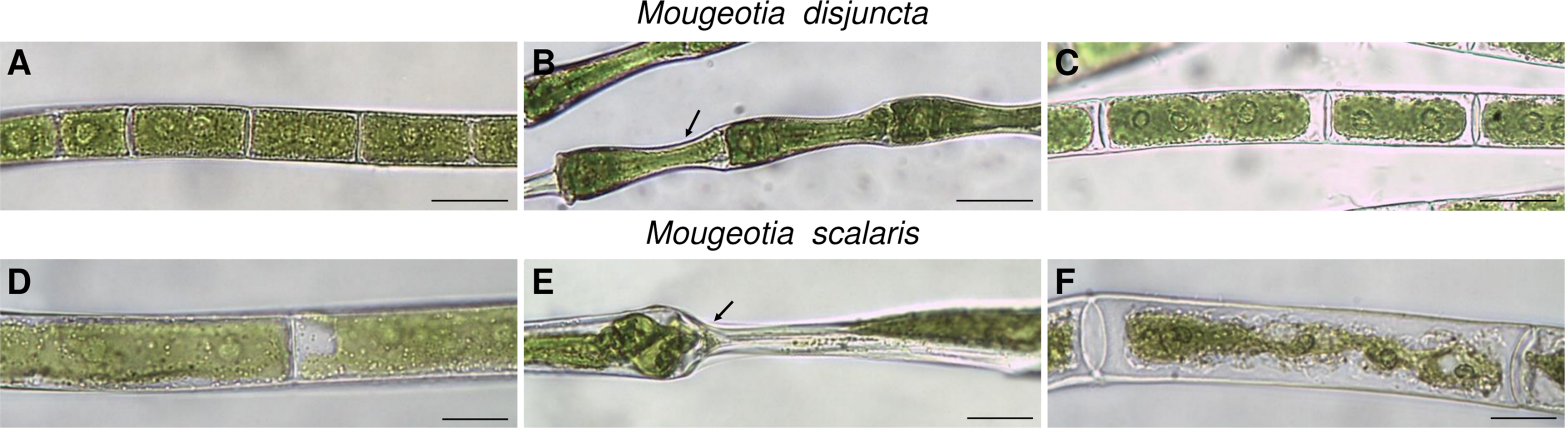
Cryo microscopical images of **(A–C)** *Mougeotia disjuncta* and **(D–F)** *Mougeotia scalaris* filaments. **(A, D)** prior ice nucleation; **(B, E)** in induced frozen state (**−**5°C), arrows indicate deformation and inward bending of the cell wall caused by freeze dehydration; **(C, F)** post freezing. Scale bars = 20 μm.

Cryo microscopy was employed to visualize cellular changes during and after freezing. *Mougeotia disjuncta* showed little visible change to the chloroplast structure after the freezing treatment when compared to controls (Figure 10A, C). The chloroplast as well as the plasma membrane of *M. scalaris*, in contrast, were clearly damaged after exposure to −5°C and ice nucleation (Figure 10D, F). When frozen, both species, exhibited clear signs of freeze dehydration with deformation and inward bending of the cell walls, while the cytoplasm was not retracted (Figure 10B, E). During this state, damages to the chloroplast in the form of tears and deformation were observed in *M. scalaris*. The cell walls of both species were able to reverse the changes caused by freeze dehydration upon thawing (Figure 10C, F).

## 4 DISCUSSION

The present study explores and compares frost hardiness and the effects of cold acclimation treatment in two *Mougeotia* species (alpine/ lowland). The investigations also include a phylogenetic analysis based on *rbc*L sequences and physiological characterization of both species, providing new data on this genus. The alpine species is possibly exposed to natural freezing events in spring when they are not covered by snow. In contrast, the lowland species is protected by the water body of the pond, from which it was isolated. This comparison provides valuable information on the adaptation capabilities of Zygnematophyceae to future climate change scenarios. The gathered data furthermore adds to the growing knowledge on the role of cold acclimation and VAZ cycle activation in the frost hardiness of streptophyte algae.

### 4.1 Phylogenetic position of *Mougeotia disjuncta* and *Mougeotia scalaris*

Zygnematopphyceae are an extremely species rich class of streptophyte algae, with the genus *Mougeotia* alone comprising 172 accepted species (Guiry, 2023). While considerable efforts have been made in sequencing species of the genus *Zygnema* (Stancheva et al., 2012; Pichrtová et al., 2018) and *Spirogyra* (Drummond et al., 2005; Chen et al., 2012; Chen & Schagerl, 2012; Stancheva et al., 2013), comparably little molecular data are available for *Mougeotia*. Moreover, traditional taxonomy and morphological species determination often proves difficult, as their sexual reproduction (conjugation) is a vital distinctive feature, yet rare or absent in many populations. To unravel the phylogenetic relationships within the class Zygnematophyceae, traditional species determination in conjunction with molecular analysis is crucial. In such manner, the present study provides *rbc*L sequences for the described *M. scalaris* (SAG 164.80) and the newly isolated *M. disjuntca* (SAG 2658), which has been determined based on its reproductive characteristics (Permann et al., 2021b). The phylogenetic analysis positioned the alpine *M. disjuncta* and the lowland species *M. scalaris* in two different clades. Interestingly, *Mougeotia* sp. (SVCK417), placed in the same clade as the alpine *M. disjuncta*, was also isolated from high altitudes (3250 m a.s.l), while *Mougeotia* sp. (SVCK240), which was closer related to the lowland *M. scalaris*, was isolated at only 6 m. a.s.l. (von Schwartzenberg et al., 2013). Despite not aim of the present study, the definition and characterization of the different clades of the genus *Mougeotia* is necessary and the *rbc*L sequences and morphological characterizations provided are valuable.

### 4.2 Habitat characterization and differences in morphology and physiology

The two *Mougeotia* species included in the present study were selected based on the differences in their natural habitats. A comprehensive morphological description, as well as temperature‐ and light responses of photosynthetic processes of these species was given. *Mougeotia disjuncta* was isolated from a single germinating zygospore, originating from the Austrian Alps (Kühtai, 2020 m.a.s.l.; Permann et al., 2021b). In this region, most Zygnematophyceae inhabit small waterbodies less than 10 cm in depth. The average annual air temperature is 0.9°C with 1653 mm of precipitation and the summer season spans from June to September (Climate, 2023). Subzero temperatures have been recorded in the months of October to May, including temperatures down to −13.7°C (January; Climate, 2023). Due to their shallow habitats and cold environmental conditions, local Zygnematophyceae most likely are exposed to low temperatures and occasional freezing events. While a recently conducted study, which analysed the seasonal dynamics of *Zygnema* mats in the Austrian Stubai Alps, near Kühtai village, recorded streamlet water temperatures ranging from −0.04 to +21.3°C during November 2018 – August 2020 and an average winter temperature of +0.9°C in 2018 and 2019 (Trumhová et al., 2023), potential freezing stress in certain microhabitats cannot be excluded. The second species included in this study, *M. scalaris*, was originally isolated from a pond at the Botanical Garden in Göttigen, Germany (700 m.a.s.l.) under snow coverage. The average annual air temperature of this German town is reported as 9.2°C with an annual rainfall of 804 mm (Climate, 2023). Subzero temperatures have been reported only for the months of December to February, with a minimum of only −1.6°C (February; Climate, 2023). Overall, the two investigated *Mougeotia* species have exhibited strikingly different environmental conditions in their natural habitats, which is hypothesized to be reflected in their frost hardiness.

Despite the simple body plan of Zygnematophyceae, both *Mougeotia* species were clearly distinguishable by their filament morphology. *Mougeotia disjuncta* was significantly (*p* < 0.001) smaller in cell width and length than *M. scalaris*. The chloroplasts of this species were also larger, which resulted in smaller‐sized vacuoles. Further TEM analyses also showed a difference in the ultrastructure of the chloroplasts, as the thylakoid membranes of *M. disjuncta* were notably denser. This was also reflected in the higher Chl amount and darker colouration of this species when compared to *M. scalaris*. While the thylakoid membranes of *M. scalaris* were more loosely arranged, gyroid cubic membranes (CM) with a moniliform appearance were observed. A complex thylakoid morphology has also been reported at the late log phase and stationary phase of cell growth in *Zygnema* filaments (Zhan et al., 2017) and in the intra‐pyrenoidal membranes of *Zygnema* zygospores (Permann et al., 2023). While in vegetative *Zygnema*, the transition from lamellar‐like thylakoid membrane morphology to gyroid CM affected the entire chloroplasts (Zhan et al., 2017), *Mougeotia scalaris*, investigated in the present study only showed an occasional formation of the moniliform superstructures. The folding of membranes and the development of more complex thylakoid morphologies might enhance the photosynthetic efficiency, similar to grana, counteracting the small chloroplast dimensions of this species.

The larger vacuoles of *M. scalaris* are suggested as contributors to the detected higher sensitivity to increased concentrations of sorbitol. The more severe plasmolysis also indicates lower concentrations of osmotically active substances in this species. As for the alpine *M. disjuncta*, full plasmolysis was reached at higher sorbitol concentrations (864 mM), when compared to *M. scalaris* (558 mM), suggesting a very negative osmotic potential and a higher tolerance against osmotic water stress. When compared to other Zygnematophyceae, the osmotic value of incipient plasmolysis of *M. disjuncta* (647 mM) was similar to two Arctic *Zygnema* strains (~600 mM) and this of *M. scalaris* (485 mM) to two *Zygnema* strains originating from the Antarctic (~300 mM; Kaplan et al., 2013).

Although isolated from different habitats, both species exhibited non‐significantly different Φ_PSII_ values (*M. disjuncta* 0.6 ± 0.0/ *M. scalaris* 0.6 ± 0.0; *p* > 0.05) under the same culture conditions, validating the further comparisons based on physiological parameters. The regression of the RLCs was similar in both species, with only the α value, the positive slope at light‐limiting photon flux rates, being significantly (*p* = 0.017) lower in the lowland *M. scalaris*. The latter indicates a lower photosynthetic efficiency under low light conditions for this species, which could be related to the larger pool of xanthophyll cycle pigments (*p* < 0.001) (Stamenković et al., 2014a). Overall, neither species showed drastic photoinhibition, as positive rETR values, even at 2,000 μmol photons m^−2^ s^−1^, were recorded. In accordance, both organisms rapidly induced photoprotective NPQ upon high light treatment (Roach & Krieger‐Liszkay, 2019). Oxygen production and consumption at increasing temperatures showed severe differences between the investigated two species. In *M. disjuncta* net photosynthesis was positive in the tested temperature range with a maximum at 35°C. In contrast, in *M. scalaris*, net photosynthesis was only positive at 5 and 10°C and negative at higher temperatures. This could be due to the fact that temperature fluctuations are smaller in a larger water body like a pond (origin of *M. scalaris*) and much higher in small pools (origin of *M. disjuncta*). Thus, we speculate that the microenvironmental conditions influence the photosynthetic performance more drastically than the elevation of their occurrence. Moreover, the higher oxygen production measured in *M. disjuncta* is associated with larger chloroplasts and tighter arrangement of thylakoid membranes observed in this species.

### 4.3 Severe differences in frost hardiness between alpine and lowland *Mougeotia* species

The freezing treatments on standard cultivated cultures revealed severe differences between the two species. The alpine *M. disjuncta* was able to retain at least 84.9 ± 27.2% (24 h after freezing experiment) of the initial Φ_PSII_ down to −5°C, with an LT_50_ of −5.8°C. Ultrastructural analysis, however, showed a slight change in thylakoid membrane arrangement after exposure to −2°C with ice formation in contrast to a treatment of −2°C, where ice nucleation was omitted. These observations suggest that ice formation does affect the internal chloroplast structure of *M. disjuncta* without impacting the physiological performance. *Mougeotia scalaris*, in contrast, showed clear ice‐susceptible properties, as cultures exposed to solely −2°C, without ice nucleation, survived this treatment (Φ_PSII_ = 106.4 ± 2.8%), but ice formation at the same temperature was lethal (Φ_PSII_ = 1.4 ± 2.4%). A clear damage of the chloroplast of *M. scalaris*, exposed to freezing events, was also observable by LM and TEM, the latter revealing a rippled structure of the thylakoid membranes.

The employment of cryo‐microscopy furthermore illustrated the morphological changes during freezing. Both species exhibited freeze dehydration in the form of deformed and bent cell walls, which was reversible upon thawing. Severe damage to the chloroplasts and the cell membranes, however, was shown only in the ice‐sensitive *M. scalaris*, while cells of *M. disjuncta* showed no visible damage. These observations and the overall higher frost hardiness of *M. disjuncta* are in accordance with the high point of plasmolysis reported for this species, as constitutively higher contents of osmotically active substances have been suggested as beneficial for freezing stress tolerance (Steiner et al., 2020).

Analogically to the present study, Trumhová et al. (2019) analysed the frost hardiness of young and mature (pre‐akinetes) *Zygnema* cultures. Very similar to the value calculated for *M. disjuncta*, an LT_50_ of −5.9°C was recorded for young *Zygnema* filaments exposed to a single freezing event. For pre‐akinetes, which occasionally even survived temperatures down to −70°C, the LT_50_ value was reported as −26.2°C. This investigated Arctic species was originally isolated in Svalbard, where the formation of pre‐akinetes, rich in storage compounds and increased cell wall thickness, is considered a key survival strategy in such extremely cold regions. A recent study conducted in Kühtai, also reported only small amounts of *Zygnema* biomass in spring, suggesting low survival rates of the filaments during winter (Trumhová et al., 2023). As the referred to study also described pre‐akinete formation in *Zygnema* populations in Kühtai at the end of the growing season, it is suggested that the cold winter conditions in the natural habitat of *M. disjuntca*, also necessitate special protective stages, which exhibit increased levels of storage compounds and cell wall thickness (Trumhová et al., 2023). Studies on *K. crenulatum* also reported additional cell wall layers and changes in organelle structure and distribution as protection against freezing stress (Steiner et al., 2020). As no pre‐akinetes have been reported in *Mougeotia*, the frequently observed conjugation and formation of zygospores in populations in the Kühtai valley (Permann et al., 2021b) might represent a crucial aspect in their adaptation to cold temperatures. Preliminary results on *Mougeotia* sp. zygospores, also isolated in the Kühtai valley, indeed suggest frost hardiness down to −11°C, exceeding that of vegetative cells (Permann, unpublished). Although no subzero temperatures were recorded in stream and lake water in the Kühtai valley in 2018–2019 (Trumhová et al., 2023), occasional freezing events are a potential stress on local Zygnematophyceae, including *M. disjuncta*. The difference in frost hardiness between the two investigated species might, therefore, be explained by their habitat conditions and specific climatic niches, as the warmer temperatures and higher water body depth most likely prevent any freezing events in the habitat of *M. scalaris*. Such a strategy has also been described for *Micrasterias denticulata* (Steiner et al., 2021) and does not require any frost hardiness or specialized cell types like zygospores or pre‐akinetes, as described above. Studies on *Cosmarium* (Zygnematophyceae) species, isolated from various habitats (including polar, tropical, alpine, and lowland), also revealed differences in ultrastructural changes upon chilling stress (+0.6°C/ 32 days), which were in accordance with their source location (Stamenković et al., 2014b). Furthermore, the initiation of programmed cell death after such prolonged cold stress was observed, which is beneficial for the survival of the algal population and a strategy that may also be employed by other Zygnematophyceae. Overall, the present study is in accordance with former investigations and shows that Zygnematophyceae, as a class, possess a great potential to acclimate to different environments, enabling them to occupy a wide range of geographic areas.

### 4.4 No enhancement of frost hardiness by cold acclimation in *Mougeotia*

While pre‐akinetes, packed with storage compounds, like lipids, can be induced by prolonged cultivation and/ or nitrogen starvation in *Zygnema* sp. (Pichrtová et al., 2014; Arc et al., 2020), a similar effect was observed in cold‐cultivated *Mougeotia*. To analyse the effects of cold acclimation on frost hardiness, both investigated species were exposed to +4°C for 3 weeks. Especially the alpine and more frost hardy *M. disjuncta* exhibited a clear increase in storage compounds after this treatment. Studies on *K. flaccidum* also reported higher numbers and sizes of starch grains in the chloroplasts, chloroplast enlargement, as well as vacuole size reduction in cold‐treated cultures (+2°C/ one week; Nagao et al., 2008). These changes were additionally accompanied by an accumulation of soluble sugars, a putative glycoside and amino acids. In *K. flaccidum* this cold acclimation process indeed resulted in a significantly enhanced frost hardiness. *Klebsormidium*, in general, has been shown to be highly resistant to both freezing and desiccation injuries (survival at −40°C; Elster et al., 2008). Regarding members of the higher branching Zygnematophyceae, Trumhová et al. (2019) also reported an increase in frost hardiness (LT_50_ = −6.5°C) in young vegetative *Zygnema* cells after a double freezing event. Studies on *M. denticulata*, which showed significant ultrastructural changes upon cold acclimation treatment (+4°C for 25 d followed by +0.5°C for 26 d in darkness), in contrast, reported no impact on their freezing stress tolerance (Steiner et al., 2021). Contradicting the first two studies and our hypothesis, no enhanced frost hardiness was found in both *Mougeotia* species. While a significant decrease in initial Φ_PSII_ was found only for *M. scalaris*, both species even showed a higher sensitivity to freezing stress. For *M. disjuncta*, a significantly decreased LT_50_ of −4.7°C was calculated. However, the negative effect was more severe in the initial hours after the experiment at +4°C, while a recovery process was observed after a period (24–50 h after the experiment) at standard cultivation conditions. Cold‐acclimated *M. scalaris* showed a more severe response to ice, as all replicates exhibited no measurable vitality right after the experiments and no signs of recovery were observed. Overall, our results indicate that *M. disjuncta* and *M. scalaris* do not respond to cold acclimation treatment with an enhancement of frost hardiness as reported for other streptophyte algae or land plants.

### 4.5 VAZ cycle activation under freezing stress

To study potential oxidative stress caused by freezing events, changes in pigment content and H_2_O_2_ production were monitored. A recent study conducted on *K. flaccidum* showed an accumulation of antheraxanthin, zeaxanthin, and total carotenes upon low‐temperature stress (5°C; Míguez et al., 2020). As an increase in frost hardiness in response to low temperatures has also been reported in this alga, it is indicated that the de‐epoxidation of xanthophylls might be involved in frost hardiness. However, the present study showed little to no changes in the pigment composition in the more frost‐hardy *M. disjuncta*, while the ice‐sensitive *M. scalaris* exhibited a clear activation of the VAZ cycle after exposure to freezing events (V_F_, X_F_). This is in accordance with the suggested correlation between xanthophyll cycle pool size and the capacity to form zeaxanthin (Demmig‐Adams, 1990), as a significantly (*p* < 0.001) larger pool of xanthophyll cycle pigments was recorded in this species. While the activation of the VAZ cycle, via the violaxanthin de‐epoxidase (VDE), has mostly been studied during light conditions (as in *K. flaccidum*), abiotic stresses, like freezing events, have recently been shown to induce de‐epoxidation also in darkness (Fernández‐Marín et al., 2018, 2021a, b). These results are supported by the present study, as all temperature experiments were conducted under dark conditions. Investigations on overwintering ferns furthermore showed an accumulation of zeaxanthin and an increase in DEPS in response to freezing (Fernández‐Marín et al., 2021a), as also observed in *M. scalaris*. This effect was shown to be reversible upon recovery after thawing but only in tolerant fern species. Similarly, the ice sensitive algal species investigated in the present study showed no signs of VAZ cycle relaxation, but an increase in zeaxanthin and DEPS was measured after thawing. As already hypothesized by Fernández‐Marín et al. (2021a) freezing‐induced VAZ cycle activation might also depend on desiccation tolerance, as VDE activation upon freezing might be in response to cellular desiccation. De‐epoxidation induced by dehydration has been reported in desiccation‐tolerant algae and bryophyte species (Fernández‐Marín et al., 2009, 2010, 2011). As the impacts of freezing stress are, partially similar to those of desiccation stress, an enhanced tolerance against desiccation might also be beneficial for dealing with freezing stress and *vice versa*. Studies on polar *Klebsormidium* have also demonstrated an enhanced desiccation stress tolerance upon cold acclimation (+5°C; Rippin et al., 2019). The lower sensitivity to water loss through osmotic stress and the exposure to semi‐terrestrial conditions in their natural habitat, indeed, suggest a higher desiccation tolerance of *M. disjuntca* compared to *M. scalaris*. The higher frost hardiness of *M. disjuncta* might also be facilitated by their smaller vacuoles, as ultrastructural changes, such as chloroplast enlargement and vacuole size reduction, have been shown to contribute to enhanced frost hardiness in *K. flaccidum* (Nagao et al., 2008). Interestingly, the more frost‐hardy species *M. disjuncta* showed no statistically relevant changes in pigment composition or DEPS upon freezing stress, excluding the de‐epoxidation of xanthophylls as a contributor to its enhanced frost hardiness. While the present study shows clear differences in VAZ cycle response in ice‐tolerant and ice‐sensitive algal species, the factors involved in this freezing‐induced zeaxanthin formation are future research avenues.

The present study also included the differentiation between freezing stress with and without triggered ice nucleation at the same temperature (−2°C). While the VAZ and DEPS change in *M. scalaris* to freezing‐thaw cycles coincides with previous studies and suggests VDE activation upon freezing, full subzero temperature cycle treatments without ice formation (X) showed no significant (*p* > 0.05) difference to X_F_. This indicates that VDE was also activated upon exposure to subzero temperatures, and not only by ice nucleation. As the samples harvested directly at the target temperature after 5 h without a warming process (V) did not display any changes in pigment content, the exposure time most likely also plays a vital role in VAZ response. Recent studies on heat stress indeed showed a logarithmic correlation between exposure duration and LT_50_ (Neuner & Buchner, 2023). Studies on *Zygnema* also reported a dependency of the severity of extracellular ice formation on temperature as well as duration of exposure at slow cooling rates (Hawes, 1990).

The nearly equal response of *M. scalaris* to X and X_F_ raises the question of whether the induced freezing events or the subzero temperature treatment caused the VAZ response after the full cycle. However, as significant zeaxanthin and DEPS increase were found in V_F_ when compared to C and V (*p* < 0.001), it can be concluded that freezing stress does activate VDE. Overall, our data demonstrate that the de‐epoxidation of xanthophylls in darkness can be triggered not only by freezing, but also by subzero temperature stress. While a correlation between F_v_/F_m_ (max. photochemical efficiencies of PSII) and DEPS was found in freezing stressed fern (Fernández‐Marín et al., 2021a), Φ_PSII_ measurements of *M. scalaris* yielded decreased values only after X_F_ but not X, which did not correspond with the aforementioned similar DEPS. While abiotic stresses also often enhance the accumulation of toxic compounds and an increase in ROS production was shown upon sudden low‐ or high‐temperature stress (Wise, 1995; Sharkey, 2005; Allakhverdiev et al., 2008), the amount of released H_2_O_2_ did not increase significantly (despite increased mean values at X) upon X_F_ or X in neither species. In higher plants, cold stress and ‐acclimation, resulting in enhanced frost hardiness, have been shown to increase the activity and level of ROS scavenging enzymatic activities (Suzuki & Mittler, 2005). Generally, the involvement of ROS in mediating cold or frost hardiness has been suggested. While, based on former data, a clear difference in H_2_O_2_ production between the frost‐hardy (*M. disjuncta*) and the ice‐sensitive (*M. scalaris*) species was expected, only the treatment X_F_ yielded significantly lower amounts (*p* = 0.006) in *M. disjuncta*. The amount of photoprotective and ROS scavenging pigments such as neoxanthin, lutein, and β‐carotene was also unaffected by subzero temperature‐ or freezing stress in *M. disjuncta*, and only elevated levels of lutein (*p* = 0.005) were measured in *M. scalaris* after V_F_. While also the VAZ cycle pigment zeaxanthin has been reported as a direct scavenger of ROS (Havaux & Niyogi, 1999; Johnson et al., 2007; Dall'Osto et al., 2010), no such correlation was found in *Mougeotia*. Overall, the level of ROS scavenging pigments was not elevated in the more frost‐hardy species and no effect of subzero temperature‐ and freezing stress on H_2_O_2_ production was found.

The reported involvement of zeaxanthin in the preservation of thylakoid membrane integrity (Havaux, 1998; Kostecka‐Gugala et al., 2003; Fernández‐Marín et al., 2013), could also not be confirmed in the present study. However, the chloroplast damage caused by freezing stress, as visualised by LM and CLMS, in *M. scalaris* might merely be too severe for this preservation mechanism. Concerning changes in Chl content, a decrease of similar extent (*p* > 0.05) in Chl *a*: *b* ratio, which was caused by decreasing Chl *a*, was detected in *M. scalaris* in response to X and X_F_ and correlated with the changes in DEPS and zeaxanthin amount. It is hypothesised that a decreasing Chl *a*: *b* ratio could be caused by a stronger PSI loss in relation to PSII, as the latter naturally exhibits a lower Chl *a*: *b* ratio (Bassi et al., 1990; Dinc et al., 2012). While the current data cannot provide information on this aspect, it is shown that degradation of only Chl *a* was triggered by subzero temperature‐ and freezing stress.

## 5 CONCLUSION

While Zygnematophyceae have been shown to tolerate a variety of abiotic stresses like UV radiation, desiccation as well as osmotic stress, little is known about the effect of freezing stress. As these streptophyte algae, however, are regularly exposed to these stresses in their natural environment and their evolutionary origin is set in the glacial late Cryogenian era, accordingly adaptation strategies are expected. To assess the extent of such mechanisms, the present study investigated the response of two *Mougeotia species* to subzero temperature‐ and freezing stress. New phylogenetic (*rbc*L) information on *M. disjuncta* and *M. scalaris* (SAG 164.80) coupled with a morphological species assignment is furthermore provided. Severe differences in frost hardiness and ice tolerance in accordance with isolation habitat (alpine/ lowland) were shown. Additionally, none of the two species was able to cold acclimate. While resistant cell types might be necessary to survive the winter season, the demonstrated frost hardiness of young vegetative cells represents a crucial survival strategy for populations exposed to sudden freezing events, which can occur throughout summer in alpine habitats. This study furthermore provides the first reports of de‐epoxidation of xanthophylls in darkness upon subzero temperature‐ and freezing stress in Zygnematophyceae. However, VDE activation does not necessarily facilitate frost hardiness and further research concerning stress‐induced zeaxanthin formation in Zygnematophyceae is needed.

## AUTHOR CONTRIBUTIONS

CP: study design, experiments (microscopy, RLC, NPQ, freezing‐experiments, H_2_O_2_, HPLC), draft ms writing, and funding; MS: study design, freezing‐experiments; TR: H_2_O_2_ and HPLC pigment analyses; VL: oxygen measurements; LAL: phylogenetic analysis; GN: study design, supervision and funding; AH: study supervision, coordination and funding. All authors have read and approved the final version of the manuscript.

## FUNDING INFORMATION

This research was funded in whole or in part by the Austrian Science Fund (FWF) 10.55776/P34181 to AH and 10.55776/P34844 to GN. For open access purposes, the author has applied a CC BY public copyright licence to any author accepted manuscript version arising from this submission. Additionally, the study was supported by an Early Stage Funding grant from the University of Innsbruck WS717006 to CP.

## CONFLICTS OF INTEREST STATEMENT

The authors declare no conflict of interest.

## Supporting information


**Appendix S1:** Supplementary Information

## Data Availability

The supporting data of the present study are available upon request from the corresponding author, Andreas Holzinger. The obtained sequence of the investigated *Mougeotia* strains are available at the GenBank under the accession numbers OR786372 (*Mougeotia disjuncta*, SAG 2658) and OR786371 (*Mougeotia scalaris*, SAG 164.80).
