# Supplementary material for: Striking differences in frost hardiness and inability to cold acclimate in two Mougeotia species (Zygnematophyceae) from alpine and lowland habitats
Source: Physiol Plant. 2024 Jan 22;176(1):e14167. doi: 10.1111/ppl.14167 (PMC10952266; doi:10.1111/ppl.14167)
Supplement: Supplementary file 1 — Appendix S1: Supplementary Information [file PPL-176-0-s001.pdf]

‘Striking Differences in Frost Hardiness and Inability to Cold Acclimate in two *Mougeotia* species (Zygnematophyceae) from Alpine and Lowland Habitats’

Charlotte Permann, Matthias Stegner, Thomas Roach, Valentina Loacker, Louise A. Lewis, Gilbert Neuner, and Andreas Holzinger

Table S1: Statistical analysis (depicting p-values) of pigment content, released hydrogen peroxide (H_2O_2), characteristic parameters of RLCs (rapid light curves), maximum NPQ (non-photochemical quenching), cell dimensions, and effective quantum yield of PSII (Φ_{PSII}) comparing *Mougeotia disjuncta* (SAG2658) and *Mougeotia scalaris* (SAG164.80). Significances were determined by two-sample t-tests. Abbreviations: A, antheraxanthin; Chl, chlorophyll; V, violaxanthin; Z, zeaxanthin.

	**Violaxanthin**	**Antheraxanthin**	**Zeaxanthin**	**AZ/VAZ**	**VAZ total**	**Neoxanthin**	**Lutein**	**β-carotene**	**Chl *a***	**Chl *b***
M. disjuncta ~ M. scalaris	0.0009	0.4256	NaN	0.0361	0.0003	0.3156	0.0206	0.6390	0.1032	0.0300
	**Chl *a*/Chl *b***	**H_2O_2**	**α**	**ETR_{max}**	**Ik**	**NPQ_{max}**	**cell length**	**cell width**	**length/width**	**Φ_{PSII}**
	0.0155	0.2479	0.0169	0.0547	0.1054	0.0946	0.0000	0.0000	0.0000	0.1833

Table S2: Statistical analysis (depicting p-values) of RLCs (rapid light curves), NPQ (non-photochemical quenching) and oxygen evolution comparing *Mougeotia disjuncta* (SAG2658) and *Mougeotia scalaris* (SAG164.80). Significances were determined by two-sample t-tests. Abbreviations: PAR, photosynthetic active radiation; P_{gross}, gross photosynthesis; P_{net}, net photosynthesis; R_d, dark respiration; T, temperature.

	**NPQ**		**RLC**		**Oxygen evolution**				
	**PAR**		**PAR**		**T**	**R_d**	**P_{gross}**	**P_{net}**	**P/R**
M. disjuncta ~ M. scalaris	0	NaN	0	0.0000	5	0.0430	0.3231	0.1523	0.1713
	617	0.7430	4	0.0004	10	0.4012	0.9857	0.8430	0.5767
	617	0.0946	29	0.0034	15	0.0271	0.4398	0.2504	0.0550
	617	0.0548	62	0.0091	20	0.1338	0.1185	0.0035	0.0037
	617	0.1049	99	0.0191	25	0.1031	0.0638	0.0015	0.0021
	617	0.8179	196	0.0273	30	0.0940	0.0807	0.0038	0.0026
	0	0.3756	361	0.2311	35	0.2998	0.1149	0.0139	0.0042
	0	0.6753	617	0.4057	40	0.3434	0.0622	0.0711	0.1014
	0	0.5374	979	0.4353					
	0	0.3799	1384	1.0000					
	0	0.1812	1661	0.6745					
	0	0.1027							
	0	0.0495							
	0	0.0091							
	0	0.0049							
	0	0.0017							

Table S3: Statistical analysis (depicting p-values) of effective quantum yield of PSII (Φ_{PSII}) in *Mougeotia disjuncta* (SAG2658) and *Mougeotia scalaris* (SAG164.80) comparing standard culture conditions ($\Phi_{PSII\ stand}$) and cold acclimation for 3 weeks at +4 °C ($\Phi_{PSIIaccl}$). Significance was determined by a two-sample t-tests.

	M. disjuncta	***M. scalaris***
**$\Phi_{PSII\ stand}$~ $\Phi_{PSII\ accl}$**	0.4079	0.0000

Table S4: Statistical analysis (depicting p-values) of released hydrogen peroxide (H_2O_2) comparing *Mougeotia disjuncta* (SAG2658) and *Mougeotia scalaris* (SAG164.80) after exposure to freezing stress at –2 °C with and without triggered ice nucleation (C, control; X, exposure to full temperature cycle; X_F, exposure to full temperature cycle + ice nucleation).

	**H_2O_2**		
	**C**	**X**	**X_F**
M. disjuncta ~ M. scalaris	0.2479	0.8716	0.0059

Table S5: Statistical analysis (depicting p-values) of pigment content and released hydrogen peroxide (H_2O_2) in *Mougeotia disjuncta* (SAG2658) and *Mougeotia scalaris* (SAG164.80) exposed to freezing stress at –2 °C with and without triggered ice nucleation (C, control; V, exposure to 5 h at target temperature; X, exposure to full temperature cycle; V_F, exposure to 5h at target temperature + ice nucleation; X_F, exposure to full temperature cycle + ice nucleation). Significances were determined by a multifactorial ANOVA analysis, followed by a Tukey's post hoc-test. Abbreviations: A, antheraxanthin; Chl, chlorophyll; V, violaxanthin; Z, zeaxanthin.

	Mougeotia disjuncta										
	**Violaxanthin**	**Antheraxanthin**	**Zeaxanthin**	**AZ/VAZ**	**Neoxanthin**	**Lutein**	**β-carotene**	**Chl *a***	**Chl *b***	**Chl *a*/Chl *b***	**H_2O_2**
**V~C**	0.6541	0.6541	NaN	0.6541	0.9653	0.5975	0.9881	0.8280	0.6800	0.6509	ANOVA: 0.211
**V_F~C**	0.9313	0.9313	NaN	0.9313	0.9962	0.9751	0.9909	0.8984	0.9914	0.4835	
**X~C**	0.6708	0.6708	NaN	0.6708	0.8126	0.9707	0.9935	0.9865	0.9773	1.0000	
**X_F~C**	0.7281	0.7281	NaN	0.7281	0.9038	0.4886	1.0000	0.9772	0.9712	1.0000	
**V_F~V**	0.9721	0.9721	NaN	0.9721	0.8550	0.3079	0.8845	0.3638	0.4408	0.9979	
**X~V**	1.0000	1.0000	NaN	1.0000	0.9908	0.9015	1.0000	0.9784	0.9349	0.6516	
**X_F~V**	0.1393	0.1393	NaN	0.1393	0.5841	0.0621	0.9746	0.5211	0.3599	0.6535	
**X~V_F**	0.9768	0.9768	NaN	0.9768	0.6236	0.7584	0.9101	0.6587	0.8494	0.4842	
**X_F~V_F**	0.3223	0.3223	NaN	0.3223	0.9841	0.8059	0.9969	0.9976	0.9998	0.4860	
**X_F~X**	0.1455	0.1455	NaN	0.1455	0.3560	0.2274	0.9841	0.8240	0.7677	1.0000	
	Mougeotia scalaris										
**V~C**	0.6482	0.6907	1.0000	0.6482	0.3349	0.8201	0.8869	0.9999	0.9990	0.9972	ANOVA: 0.0992
**V_F~C**	0.0000	0.8213	0.0000	0.0000	0.1172	0.0045	0.9740	0.0612	0.3884	0.9964	
**X~C**	0.0000	0.0001	0.0000	0.0000	0.9843	0.2088	0.0327	0.0001	0.8672	0.0000	
**X_F~C**	0.0000	0.0003	0.0000	0.0000	0.7351	0.4043	0.8242	0.0002	0.6424	0.0000	
**V_F~V**	0.0000	0.9990	0.0000	0.0000	0.9408	0.0221	0.9972	0.0484	0.2816	1.0000	
**X~V**	0.0000	0.0002	0.0000	0.0000	0.5949	0.7096	0.1325	0.0001	0.7460	0.0000	
**X_F~V**	0.0000	0.0016	0.0000	0.0000	0.9361	0.9321	0.9999	0.0001	0.5019	0.0000	
**X~V_F**	0.0000	0.0002	0.0000	0.0000	0.2456	0.1567	0.0813	0.0089	0.8851	0.0000	
**X_F~V_F**	0.0000	0.0012	0.0000	0.0000	0.5815	0.0733	0.9886	0.0118	0.9880	0.0000	
**X_F~X**	0.9696	0.5594	0.9727	0.9696	0.9469	0.9853	0.1645	0.9997	0.9911	0.8766	
